# High-Ranking Geladas Protect and Comfort Others After Conflicts

**DOI:** 10.1038/s41598-018-33548-y

**Published:** 2018-10-16

**Authors:** Elisabetta Palagi, Alessia Leone, Elisa Demuru, Pier Francesco Ferrari

**Affiliations:** 1 Museo di Storia Naturale, Università di Pisa, Via Roma 79, 56011 Calci - PISA, Italy; 2 Institut des Sciences Cognitives Marc Jeannerod, CNRS/Université Claude Bernard Lyon 67 Bd Pinel, 69675 Bron Cedex, France

## Abstract

Post-conflict affiliation is a mechanism favored by natural selection to manage conflicts in animal groups thus avoiding group disruption. Triadic affiliation towards the victim can reduce the likelihood of redirection (benefits to third-parties) and protect and provide comfort to the victim by reducing its post-conflict anxiety (benefits to victims). Here, we test specific hypotheses on the potential functions of triadic affiliation in *Theropithecus gelada*, a primate species living in complex multi-level societies. Our results show that higher-ranking geladas provided more spontaneous triadic affiliation than lower-ranking subjects and that these contacts significantly reduced the likelihood of further aggression on the victim. Spontaneous triadic affiliation significantly reduced the victim’s anxiety (measured by scratching), although it was not biased towards kin or friends. In conclusion, triadic affiliation in geladas seems to be a strategy available to high-ranking subjects to reduce the social tension generated by a conflict. Although this interpretation is the most parsimonious one, it cannot be totally excluded that third parties could also be affected by the negative emotional state of the victim thus increasing a third party’s motivation to provide comfort. Therefore, the debate on the linkage between third-party affiliation and emotional contagion in monkeys remains to be resolved.

## Introduction

Conflicts in social animals can have various immediate and long-term outcomes. Immediately following a conflict, opponents may show a wide range of responses, from tolerance and avoidance of open conflict, to aggression^1^. Due to the potential negative consequences of agonistic interactions, such as renewed hostility towards the victim and conflict spreading across the social group, conflict management strategies are thought to have evolved to prevent and repair damage following conflicts that could be disruptive to the social group^2^. From an ecological point of view, conflicts can also lead to negative consequences in foraging activities, with victims spending less time searching for food because they are either excluded from access to resource or spend more time engaged in social vigilance^3^.

Among post-conflict behaviors, reconciliation is the main form of conflict *resolution*. De Waal & van Roosmalen^4^ first defined reconciliation as the tendency of aggressor and victim to contact each other shortly after a conflict and engage in affiliative behavioral patterns. In the subsequent decades, many studies have been devoted to exploring the dynamics and functions of the conciliatory mechanism^5–8^. The main advantage of reconciliation process is to end hostilities and restore the relationship between the opponents. When the aggressor and the victim share a good relationship or are kin, the probability of a conciliatory contact increases and consequently, so do the benefits derived from conflict *resolution*^3^. However, reconciliation can also entail a certain level of risk especially for the victim because approaching a former aggressor immediately after the fight can lead to a further attack^9^. Reconciliation may thus be absent or infrequent when aggression occurs in highly competitive contexts (e.g., feeding), if the intensity of the conflict is severe^7,10^ or if the conflict is highly directional^11,12^.

Dyadic reconciliation, however, is not the only post-conflict affiliative interaction available to limit the damage caused by aggression. Triadic post-conflict affiliation has been described in several primate (reviewed in^13–15^) and non-primate species (ravens, *Corvus corax*^16^; dogs, *Canis familiaris*^17^; wolves, *Canis lupus*^18^; prairie voles, *Microtus ochrogaster*^19^). Triadic post-conflict affiliation refers to affiliation between the two opponents and an uninvolved bystander (i.e. a ‘third party’). Such contacts may involve either aggressors or victims, and the third parties may be kin to either one of the opponents or unrelated to them^20–23^. The function of such contacts presumably varies according to the identity of the third party and whether the affiliation is directed to the aggressor or the victim^13^. Third party affiliation can be provided spontaneously (i.e., non-solicited or spontaneous triadic affiliation) or following a request for such affiliation by either the aggressor or the victim (i.e., solicited triadic affiliation)^15–17,24^.

Third party spontaneous affiliation towards the victim has been observed in a variety of mammalian and non-mammalian species (chimpanzees, *Pan troglodytes*^4,25–32^; gorillas, *Gorilla* spp.^33–35^, bonobos, *Pan paniscus*^24,36–38^, humans, *Homo sapiens*^39^, wolves^18,40^, ravens^16^, rooks^41^ and horses^42^, prairie voles^19^). Similar to dyadic reconciliation, the occurrence and frequency of third party affiliation is affected by several factors, such as the presence/absence of reconciliation, the relationship quality between the victim and the third party, the history of previous agonistic interactions and the level of redirection of a group (redirection is when a victim redirects aggression against a bystander)^3^. All these factors can affect the outcome of triadic post-conflict affiliation and do so in different ways depending on the species and the relationships of the particular individuals being observed.

When third party affiliation is spontaneously offered to socially bonded subjects (i.e., kin/friends) and significantly reduces anxiety in the victim, it can be labeled as consolation. Anxiety can be defined as an emotional state deriving from motivational conflict that can be induced by conditions of uncertainty^43^. If not resolved, the heightened level of anxiety can lead to reduced maintenance activities and altered social interactions by the victim, which, in turn, can have a negative impact on other group members.

In primates, consolation has been demonstrated in humans^44^, chimpanzees^31,32^ (although one study did not find evidence in this species^29^), bonobos^24,37,38^ and Tonkean macaques (*Macaca tonkeana*)^45,46^. It is interesting to note that consolation was not found in several macaque species whose social groups are based on more despotic relationships (*Macaca fascicularis*, *M*. *fuscata*, *M*. *sylvanus*, and *M*. *nemestrina*^25^). In despotic macaques, the distribution of affiliative dyadic interactions (e.g., grooming) is determined by rigid hierarchical and nepotistic rules^47^. In these species, the investment in creating and maintaining social bonds among unrelated subjects is extremely low and mainly focused on with high-ranking subjects^47^. The presence of consolation in the more tolerant *Macaca tonkeana* is not surprising given the extremely high levels of social bonding that extends beyond kin and more flexible dominance relationships^46^.

Geladas (*Theropithecus gelada*) live in what are referred to as multi-level societies^48^. The basic element of these societies consists of a One-Male Unit (OMU), a unit normally containing one adult male, six to eight adult females and their offspring. Some units may contain more than one adult male, but only one male typically copulates with the females. Several OMUs may live closely together forming a higher level of social organization. Depending on the degree of association among the constituent OMUs, such units are called “teams” if they associate regularly or “bands” if they associate less regularly^49–51^. Typically, adult males dominate females and the males from the different OMUs avoid interacting with the females belonging to other OMUs^52,53^. Geladas are characterized by female philopatry and male dispersal. The strongest social relationships within an OMU are among the related females (high level of agonistic support, embracing and grooming), with the males engaging in friendly interactions with some the of those females^54^. Hamadryas baboons (*Papio hamadryas*) also form social groups based on OMUs but these are maintained by the males aggressively herding the females. In contrast, OMU integrity in geladas results from the strong bonds among group members^55^. The feeding ecology of geladas probably explains why this species shows a tolerant dominance hierarchy and high levels of affiliation. Geladas’ diet is mainly based on grass, an abundant resource, which is impossible to monopolize. This can lead to a low level of competition among group members and reduced differences in foraging afforded by differences in social rank^48,56^. Geladas are considered a socially tolerant species, a characterization reflected in the use of coalitionary support in favor of the victims of aggression^57^ and by the frequent occurrence of affiliative behaviors (e.g., grooming, embracing, alloparental care) among unit members, especially among the females^57–59^.

Given the importance of social relationships in geladas, group cohesion could easily be disrupted by unresolved conflict, making conflict *resolution* particularly important in maintaining group integrity. The first study demonstrating the presence of dyadic reconciliation in geladas showed that the behavior occurs in the first two minutes after the occurrence of aggression^60^, a finding later confirmed by Leone and Palagi^61^. Because of the presence of reconciliation and their high levels of cohesiveness and tolerance, geladas are good candidates to test specific hypotheses on the potential roles of third party affiliation towards the victim, including the consolatory function.

Many hypotheses have been formulated to explain the existence/evolution of triadic post-conflict affiliation. The *Self-Protection Hypothesis* predicts that triadic affiliation protects the third party from becoming the victim of redirection^32,62–64^. Redirection occurs when the victim immediately attacks another subject not involved in the previous conflict^14,23^. In this perspective, redirection should be common and affiliation should be received primarily from those individuals that are frequently the target of redirection and from the individuals ranking lower than the victim (presumably more at risk). If the *Self-Protection Hypothesis* applies to geladas, we expect that the probability of redirection should be reduced as a result of third party affiliation.

Reconciliation between former opponents restores the relationship jeopardized by the conflict and reduces distress both in the victim and in the aggressor^3^. The *Substitute for Reconciliation Hypothesis*^5^ predicts that third party affiliation towards the victim can also play a role in reducing its anxiety. According to this hypothesis, we expect that geladas show third party affiliation toward the victim more frequently in the absence of reconciliation.

According to the *Victim Protection Hypothesis* third party affiliation protects the victim against further attacks thus providing direct benefits for the victim. This could be extremely important not only for the victim itself, but also to limit the diffusion of aggression across the group. The latter would be important in maintaining cohesion. If so, we expect that third party affiliation would significantly reduce the probability of renewed attacks on the recipient of previous aggression.

The *Consolation Hypothesis* predicts that third party affiliation is primarily received from a victim’s closely bonded/related partner and that such affiliation reduces the victim’s anxiety. In primates, changes in anxiety level can be assessed by scoring the occurrence of self-directed behaviors, such as scratching^65–70^. According to the empathic gradient hypothesis^71^, subjects sharing strong affiliative bonds are more prone to engage in consolatory contacts. Moreover, the victim’s anxiety would decrease because closely bonded partners (kin and friends) are supposed to be more effective in relieving anxiety, due to the good relationship quality shared with the victim^25,30,64,72^. If consolation is the driving mechanism of third party affiliation in geladas, we expect that it should be biased towards kin/friends and effective in reducing victim’s anxiety.

The aim of the present study was to test these hypotheses in captive geladas involving long-term data collection focused on triadic post-conflict mechanisms. Although the hypotheses are tested and discussed separately, they are not mutually exclusive, with different combinations of benefits possible among individuals with different relationships.

## Results

The colony of geladas, subject of the study, was made up of two One-Male Units (OMUs). Across the four years of observation the two OMUs were composed of 47 subjects including all age-class combinations. The exact composition of each group including the age of each subject in the years observed is provided in Supplementary Table 1.

### Demonstration and quantification of the phenomenon

We gathered behavioral data through the Post-Conflict/Matched-Control method [PC-MC^20^] resulting in 671 PC-MC pairs (see the Methods for details). To demonstrate the presence of spontaneous triadic post-conflict affiliation, we considered only the subjects with at least eight PC-MCs during in which reconciliation did not take place. For each animal we determined the number of three types pairings. In ‘attracted’ pairs affiliative contacts occurred earlier in the PC than in the MC (or they did not occur at all in the MC). In ‘dispersed’ pairs the affiliative contacts occurred earlier in the MC than in the PC (or they did not occur at all in the PC). In ‘neutral’ pairs affiliative contacts did not occur in any of the two conditions (PC and MC) or both occurred in the same minute after the ending of the conflict.

**Figure 1:**
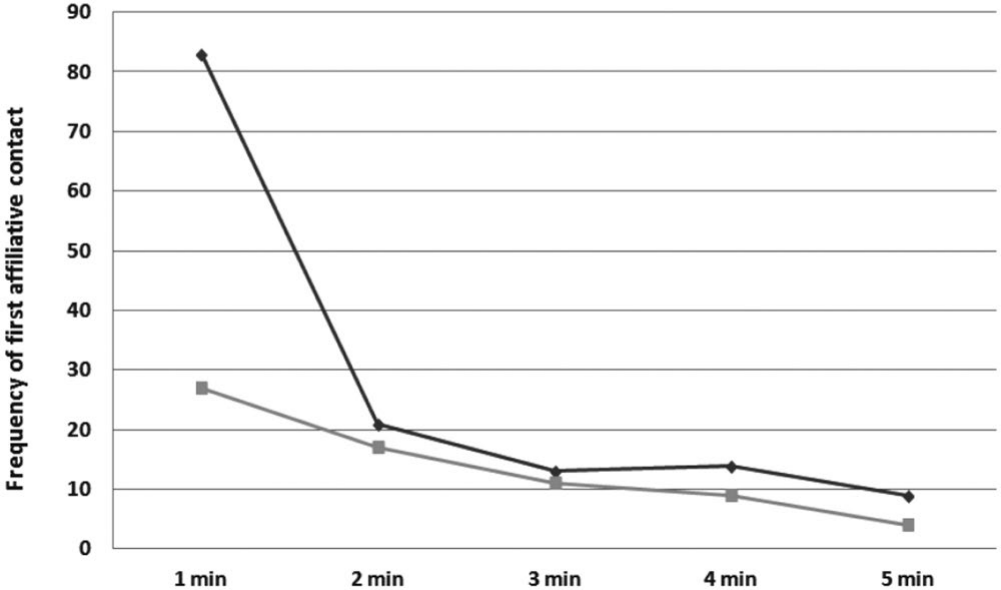
Temporal distribution of first affiliative contacts in PCs (dark grey) and MCs (light grey) for unsolicited post-conflict affiliation. Frequencies of first affiliative contacts are depicted on the Y axis. The phenomenon was present only in the first minute of observation (T_1min_ = 0.00; n = 17; ties = 0; p = 0.0001; T_2min_ = 5.00, n = 17, ties = 3; p = 0.502; T_3min_ = 2.00; n = 17, ties = 11, p = 0.688; T_4min_ = 1.00; n = 17; ties = 11, p = 0.188; T_5min_ = 1.00, n = 17, ties = 10, p = 0.281).

Geladas engaged in spontaneous triadic affiliation towards the victim (for an operational description of spontaneous triadic affiliation see the Method section). The rates of third party post-conflict spontaneous affiliation were higher in PCs than in MCs for the first minute of the post-conflict period (attracted pairs >dispersed pairs; Wilcoxon’s T_1min_ = 0.00; n = 17; ties = 0; p = 0.0001; with Bonferroni correction p < 0.01; TCT_1min_ = 26.49% ± 3.98 SE; Fig. 1).

To demonstrate solicited triadic post-conflict affiliation (see the Method section), we considered only the subjects with at least eight PC-MCs during which reconciliation did not take place. We did not find any evidence for the presence of solicited triadic affiliation in our study group. The Wilcoxon signed-rank test did not show any significant difference between attracted and dispersed pairs (Wilcoxon’s T_1min_ = 6.00; n = 19; ties = 5 p = 0.187; Wilcoxon’s T_2min_ = 6.00; n = 19; ties = 8; p = 0.464; Wilcoxon’s T_3min_ = 4.00; n = 19; ties = 10; p = 0.516; Wilcoxon’s T_4min_ = 7.00; n = 19; ties = 10; p = 0.148; Wilcoxon’s T_5min_ = 5.00; n = 19; ties = 10; p = 0.973).

Due to the absence of solicited triadic affiliation all further analyses were focused on spontaneous triadic affiliation. We measured the ‘Triadic Contact Tendency’ (TCT) defined as the number of attracted minus the number of dispersed pairs divided by the total number of PC–MC pairs (see Methods for further details). The most frequent patterns used by animals when engaging in spontaneous triadic affiliation were playing, affiliative body interactions (touching/embracing) and affiliative facial expressions and vocalizations (lip-smacking/grunting + moan). Playing (mean TCT_play_ = 42.71 ± 17.61 SE), body interactions (TCT_touching/embracing_ = 23.53 ± 11.02 SE) and affiliative facial expressions and vocalizations (TCT_lip-smacking/grunting+moan_ = 54.76 ± 16.98 SE) were significantly higher during the first minute of PCs than during the first minute of MCs (Wilcoxon’s T_playing_ = 9.00; n = 16; ties = 4; p = 0.018; Wilcoxon’s T_touching/embracing_ = 14.00; n = 19; ties = 6; p = 0.028; Wilcoxon’s T_lip-smacking/grunting+moan_ = 10.00; n = 14; ties = 1; p = 0.011, with Bonferroni correction p < 0.02). On the other hand, we did not find any difference between attracted and dispersed pairs for contact sitting (Wilcoxon’s T_contact sitting_ = 31.50; n = 17; ties = 2 p = 0.115) and grooming (Wilcoxon’s T_grooming_ = 20.00; n = 14; ties = 4; p = 0.590).

### Self-Protection Hypothesis

Of the 29 different victims of aggression, only six individuals showed redirection. The mean level of redirection calculated on the six subjects indicates that the phenomenon was rare (1.31% ± 0.38 SE).

### Victim Protection Hypothesis

**Figure 2:**
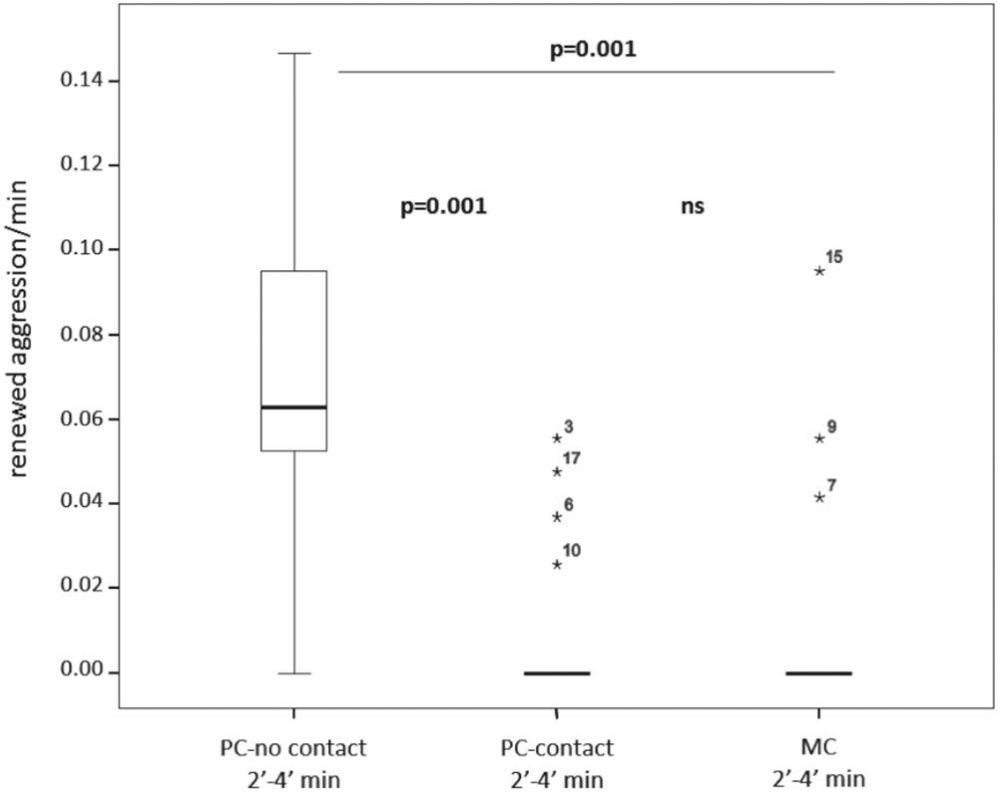
Boxplots showing the rate of renewed aggression per minute of observation (2–4 min) in absence of postconflict third-party affiliation (PC-no contact; in absence of reconciliation), in presence of post-conflict third-party affiliation (PC-contact; in absence of reconciliation) and in absence of conflict (MC). The box plots show the median and 25^th^ and 75^th^ percentiles; the numbered whiskers indicate the outlier values within 1.5 times the interquartile range, IQR. N = 18.

The rate of renewed conflicts towards the victim in absence of reconciliation and third-party affiliation was significantly higher in PC compared to MC in the first four minutes after the aggression (Wilcoxon’s T_1min_ = 14.50; ties = 3; N = 27; p = 0.0001; Wilcoxon’s T_2min_ = 0; ties = 9; N = 27; p = 0.0001; Wilcoxon’s T_3min_ = 12; ties = 11; N = 27; p = 0.004; Wilcoxon’s T_4min_ = 18; ties = 12; N = 27; p = 0.017; Wilcoxon’s T_5min_ = 17; ties = 16; N = 27; p = 0.154; with Bonferroni correction p < 0.01; Supplementary Fig. 1) To understand the effect of triadic affiliation in absence of reconciliation, we selected the 2–4 min time window for the analysis of renewed aggression. In the following analysis, the 1 min was excluded because it was characterized by the presence of the triadic affiliation (Fig. 1). The 2–4 min time window was selected because in this period the rate of PC renewed aggression remained significantly higher than the rate of aggression recorded in the same time window in the MC. The 5 min was excluded because the rates of aggression did not differ between the PC and the MC. The frequency of renewed aggression significantly differed across the three conditions: without spontaneous third-party affiliation (PC-no contact), following spontaneous third-party affiliation (PC-contact), and matched-control (MC) (Friedman’s Chi-square = 20.414, N = 18, df = 2, p = 0.0001). We found that renewed aggression in PC-no contact were significantly more frequent than those in PC-contact (post-hoc Dunnett’s test: q = 5.00, p < 0.001) and in the MC (PC-no contact vs MC contact: Dunnett’s test: q = 5.18, p < 0.001). Finally, we did not find any significant difference between renewed aggression in PC-contact and MC (Dunnett’s test: q = 0.47, ns; Fig. 2) (with Bonferroni correction p < 0.02).

### Substitute for Reconciliation Hypothesis

Third-party affiliation was more frequent in absence of reconciliation than in its presence (Wilcoxon’s T = 8.00; ties = 1; N = 29; p = 0.0001).

### Consolation Hypothesis

**Figure 3:**
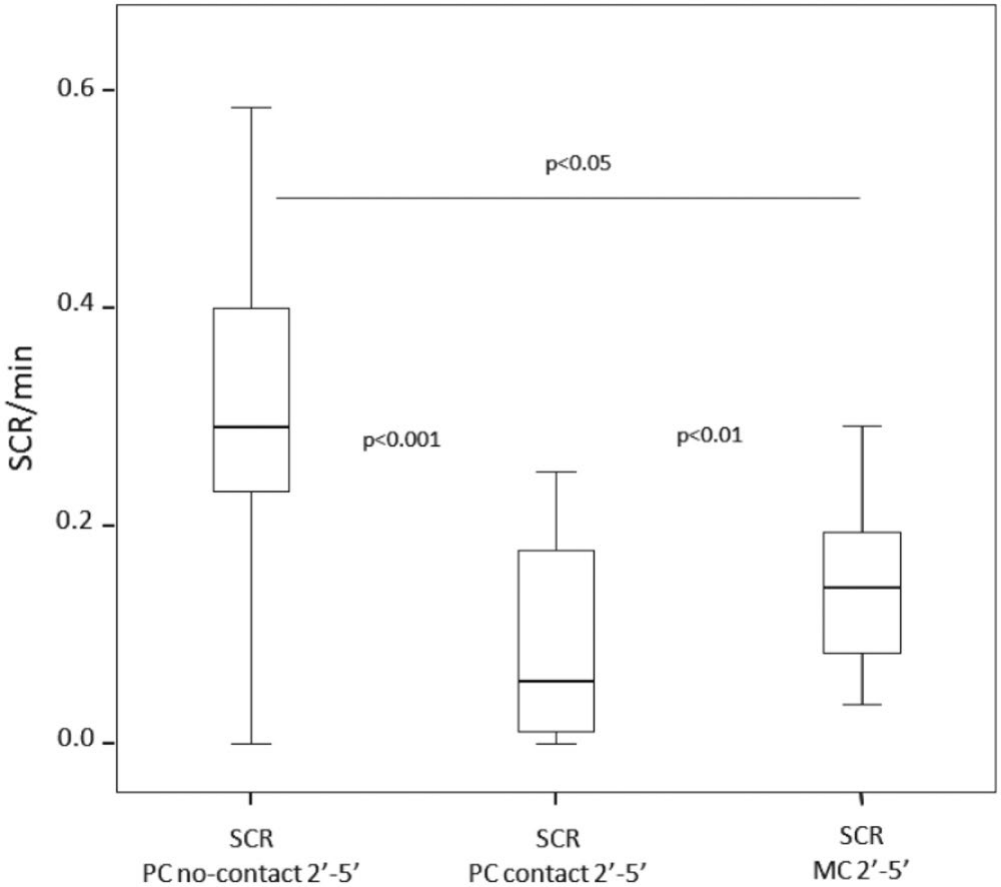
Boxplots showing the scratching per minute of observation (2–5 min) in absence of postconflict third-party affiliation (PC-no contact; in absence of reconciliation), in presence of post-conflict third-party affiliation (PC-contact; in absence of reconciliation) and in absence of conflict (MC).

Scratching rates during PCs-no contact were significantly higher than those during MCs across a 4-min time window (2–5 min), therefore the analysis of scratching in presence of spontaneous third-party affiliation was limited to that time-window (Supplementary Fig. 2). Scratching rates were significantly different across all the three conditions: without spontaneous third-party affiliation (PC-no contact), following spontaneous third-party affiliation (PC-contact), and matched-control (MC) (Friedman’s Chi-square = 12.52, N = 22, df = 2, p = 0.001). We found that scratching rates in PCs-contact were lower than those in MCs (post-hoc Dunnett’s test: q = 2.81, p < 0.01). Both scratching rates in PCs-contact and in MCs were significantly lower than those recorded in PCs-no contact (PC-contact vs PC-no contact: Dunnett’s test: q = 3.48, p < 0.001; MC vs PC-no contact: Dunnett’s test: q = 2.11, p < 0.05) (Fig. 3) (with Bonferroni correction p < 0.02).

**Figure 4:**
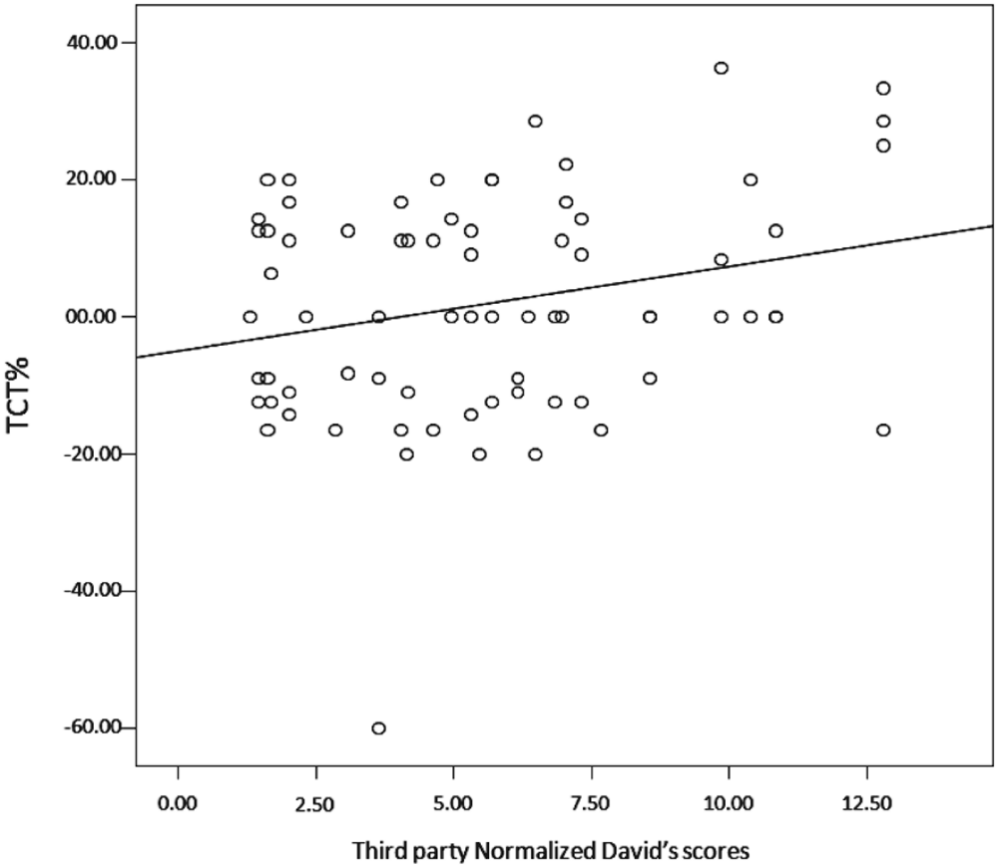
Scatter plot showing the positive correlation between TCT% values and Third-party Normalized David’s scores (R^2^ = 0.06).

To verify which factors (Table 1) affected the Triadic Contact Tendency (TCT) levels between victims and third party, we ran a Linear Mixed Model (LMM). We tested models for each combination involving the variables of interest, spanning from a single-variable model to a model including all the fixed factors (full model). All possible variable combinations were tested. The best model included the interaction of kinship with bonding (“kinship*bonding_gr_”), the sexes forming the dyad (sex_combination_), the age of the subjects forming the dyad (age_combination_), the rank of the victim (Normalized David’s Score_victim_) and the rank of the third-party (Normalized David’s Score_third-party_) (for the best model Akaike’s Corrected Information Criterion, AICc = 535.92; for the next-best model Akaike’s Corrected Information Criterion, AICc = 550.08; ΔAIC = 14.16) and explained about 94.18% of the distribution (see Table 2 for the AICc of the second and third model tested; AICc of the intercept = 612.318). Within this model only Normalized David’s Score_third-party_ was a statistically significant factor with high-ranking individuals providing more spontaneous contacts than low ranking individuals (Table 3; Fig. 4).

**Table 1:** Description of variables used in LMM analyses. NDS = Normalized David’s Scores.

NAME	TYPE
**Dependent Variable**	
*TCT*	Scale (Triadic Contact Tendency)
**Fixed Explanatory variables**	
*Individual characteristics*	
Sex Combination	Nominal (00 = male-male; 01 = male-female; 10 = female-male; 11 = female-female)
Age Combination	Nominal (11 = adult-adult, 00 = immature-immature, 10 = adult-immature, 01 = immature-adult)
NDS_VICTIM_	Scale
NDS_THIRD-PARTY_	Scale
*Relational characteristics*	
Kinship	Dichotomous (1 = kin, 0 = non-kin)
*Affiliation levels*	
Social bonding (grooming)	Ordinal (0 = week, 1 = medium, 2 = strong)
**Random Variables**	
VictimID*Third-PartyID	Nominal

**Table 2:** Table showing the fixed variables included in the three models showing the lowest values of the Akaike Corrected Information Criterion (AIC_C_).

Random effects	Fixed effects	AICc	Δ AICc	EXP(−0.5 * Δi)	Wi
VictimID*Third-PartyID	Kinship*Bonding_gr_;NDS_victim_;NDS_third-party_;SEX_combination_;AGE_combination_	535.92	0.00	1.00	0.94
	Bonding_gr_; NDS_victim_;NDS_third-party_;SEX_combination_;AGE_combination_;Kinship	550.08	14.16	0.03	0.03
	Bonding_gr_;NDS_third-party_;SEX_combination_;AGE_combination_;Kinship	551.89	15.97	0.02	0.02

The difference between the AIC of the best model and the AIC of each other model (ΔAICc) and Akaike Weight (Wi) are also reported. NDS = Normalized David’s Scores.

**Table 3:** Best LMM explaining the frequency of TCT (AICc = 535.920).

TCT (AICc = 547.874)	F	df1	df2	Sig.
**Fixed Explanatory Variables**				
Intercept	0.855	13	59	0.602
SEX combination	0.700	3	59	0.556
AGE combination	0.321	3	59	0.810
KIN*BONDgr	0.816	5	59	0.543
NDS_VICTIM_	0.132	1	59	0.718
NDS_THIRD-PARTY_	5.981	1	59	0.017
**Random variables**	**Variance**			
Victim*Third-party ID	1.013			0.311

## Discussion

To understand why and how animals respond to agonistic interactions provides valuable insights into the evolution of socio-cognitive and emotional capacities, especially if comparative studies are conducted by applying the same methodological approaches across different species^18,30,45,57,63^. The presence of unsolicited triadic affiliation in geladas permitted the testing some hypotheses about the potential functions of such social behavior. The findings from this study could then be compared to those from other primate and non-primate species. Our data do not support the *Self-Protection* hypothesis, but do provide support for the *Victim Protection* and the *Substitute for Reconciliation* hypotheses. As only two of the three criteria defining consolation were met, our findings cannot draw any clear-cut conclusions about the *Consolation* hypothesis. In the following sections we discuss our findings with regard to these hypotheses and their implications for a better understanding of the functions of post-conflict affiliation in geladas.

Geladas engaged in unsolicited triadic affiliation towards the victim in the first minute after a conflict (Fig. 1) and this is consistent with the results from several other primate^35,36,45,72^ and non-primate species^18,19,41^. The most surprising result is the absence of the solicited version of triadic contacts. Interestingly, while both spontaneous and solicited triadic contacts are present in bonobos, only spontaneous triadic contacts lead to the relief from distress in the victim^24^. This result underlines the functional dichotomy of spontaneous and non-spontaneous post-conflict affiliation. It seems that also in geladas the spontaneity of the contact is a key factor in triadic post-conflict affiliation.

In geladas, reconciliation^60,61^ and third party affiliation are both present, at least in captive populations. Our results show that when reconciliation fails to occur, the frequency of third party affiliation increases (supporting the *Substitute for Reconciliation* hypothesis). Detecting general patterns of post-conflict affiliative contacts is relatively easy, but parsing out the proximate and ultimate causes of post-conflict mechanisms is more challenging given the strong dependence of affiliative interactions on a wide range of variables related both to the social context and to the variability in the types of affiliative contacts preferentially used by different individuals^4,27,29,73,74^. Leone and Palagi^61^ demonstrated that geladas living in the same colony did not show any preference in the behavioral pattern used to reconcile (e.g., grooming, contact sitting, lip-smacking/vocalizations). On the contrary, our findings suggest that bystanders selectively engaged in short play bouts, affiliative body interactions (touching/embracing) and facial expressions and vocalizations (lip-smacking/grunting + moan) to affiliate with the victim. Therefore, although third party affiliation can function as a substitute for reconciliation, these two conflict management phenomena are expressed differently. Play was generally preferred by juveniles/subadults, even though adults also exchanged some playful contacts. A similar result was also found in gorillas^34^. In this species the immature animals, who were the best “consolers”, engaged in short play bouts with the victims to affiliate with them^34^. In geladas, third parties preferred engaging in rapid behavioral patterns, such as play, facial expressions and vocalizations, possibly because of the high level of arousal of the performers in these post-conflict contexts. Social grooming and sitting closely together with bodily contact tend to occur under more relaxed conditions.

Many authors describe triadic, post-conflict affiliation as an investment from which both victims and bystanders should gain direct and/or indirect benefits^3,36,75–77^. Given that among group living primates, victims can redirect aggression towards others, it is even possible to hypothesize that bystanders should avoid interacting with the recipient of aggression^78,79^. This is not the case of geladas, where triadic post-conflict interactions do not seem to be so risky given the extremely low levels of redirected aggression. In this view, the *Self Protection* hypothesis cannot explain our results. *Macaca tonkeana*, a species sharing with *Theropithecus gelada* tolerant social relationships and third party affiliation, also lacks redirection^45^. In contrast, other species of the *Macaca* genus, which have more despotic social relationships, show high levels of redirection and the absence of third party affiliation^45,80–82^. Our results also contrast with the function of spontaneous third-party affiliation found in mandrills (*Mandrillus sphinx*), a more despotic species^63,83^. Schino and Marini^63^ found that victims received most affiliation from those bystanders that were frequently the target of redirection and that bystander affiliation reduced the probability of redirected aggression. Therefore, in mandrills triadic post-conflict affiliation functions as a self-protection strategy, as the third-party can gain an immediate benefit. It appears, therefore, clear that post-conflict triadic affiliation works differently depending on the social style of the species.

From the victim’s perspective, receiving spontaneous triadic affiliation can provide short-term benefits. We found that after the triadic contact, the scratching rates of the victim significantly decreased, thus demonstrating that this kind of affiliation can reduce anxiety in the victim (Fig. 3). Moreover, triadic post-conflict affiliation protects the victim against further attacks, as the frequency of renewed aggression decreased after third-party interactions had occurred (supporting the *Victim Protection* hypothesis) (Fig. 2). A previous study carried out on the same colony of geladas showed that the probability of renewed aggression on the victim was not significantly affected by the presence of reconciliation^61^. These differing findings suggest that spontaneous third party affiliation and dyadic reconciliation can have different functions, leading to different social outcomes.

Even though the *Victim Protection* hypothesis has been rarely tested, third party affiliation has now been shown to protect victims in four primate species (chimpanzees^28^, bonobos^24^, Tonkean macaques^45^, geladas in the present study). As far as we know, only two studies have explicitly tested this hypothesis in species of monkeys with differing social styles. Third party affiliation in the egalitarian Tonkean macaque protects the victim from further attacks^45^, whereas such effect was not found in mandrills^63^, a species with a more despotic social style^83^. The apparently contrasting results deriving from these studies are in line with the different social dynamics characterizing the two species and, more specifically, with their different levels of tolerance *sensu*^84^.

The distribution of the triadic consolatory tendency (TCT) in our study was significantly affected by the rank of bystanders, although other factors also converged in explaining the best model (see Tables 2 and 3). The highest-ranking bystanders provided the highest levels of triadic affiliation to the victim (Fig. 4). In the same study group, we found that dominance rank also played a role in agonistic support, which was mainly provided by high-ranking individuals to the victims of the ongoing aggression^57^. In geladas, supporting the victim during the conflict and providing protection and comfort when the conflict ceased, may be driven by the so-called ‘community concern’, a pacifying motivation to maintain the stability of the group which is beneficial for all group members^57,85,86^. Therefore, triadic affiliation and agonistic support could be viewed as two sides of the same coin, providing protection to the victim and, consequently, bringing benefits to the whole group.

**Figure 5:**
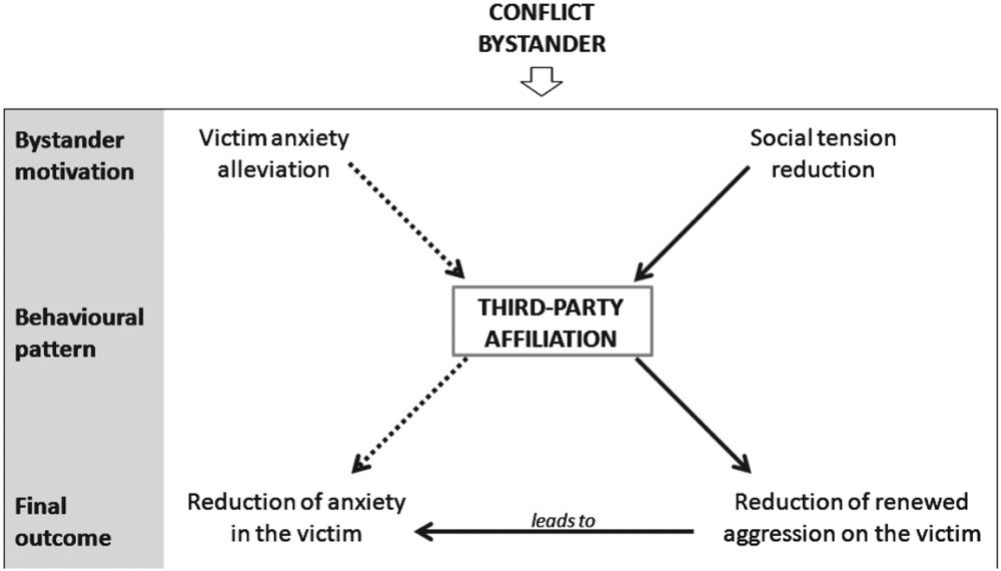
The model we proposed on the basis of our findings on geladas. For the explanation see the Discussion.

The effects of triadic affiliation on reducing anxiety and lowering the probability to be the target of renewed aggression, are two direct benefits gained by the victim. But the finding that affiliation was not biased toward either kin or friends, supports that the main benefit for the third party involved is related to group stability and cohesion. Based on our data, there are two possible, but not mutually exclusive, explanations linked to the motivations driving the third-party towards the victim (Fig. 5). Firstly, the third-party could be affected by the behavioural arousal shown by the victim and be motivated to reduce it by providing comfort. Secondly, the motivation leading the third-party to affiliate with the victim is the reduction of renewed aggression. By protecting the victim against potential renewed aggression, the third party’s intervention can break the cycle of aggression^57^. This may be especially true, if as suggested by our data, that affiliation is provided by the highest-ranking members of the group. In geladas^57^, as in many other primate species^14^, highest-ranking individuals play an important role in conflict management because their interventions are more effective in maintaining peaceful relationships^57^. The affiliative contact offered by high-ranking third party animals is most effective in reducing the likelihood of renewed aggression towards the victim as well as their level of anxiety.

The reduction of victim’s anxiety together with a bias towards kin and friends are the two main prerequisites that must be satisfied to consider spontaneous third-party affiliation as consolation. Therefore, our data do not explicitly support the *Consolation* hypothesis, even though they support the *Victim Protection* and the *Substitute for Reconciliation* hypotheses. We want to emphasize that the reduction of victim’s anxiety could also be a by-product deriving from a conflict management strategy enacted by high-ranking individuals to reduce further aggression (Fig. 5).

To determine the motivation driving subjects to engage in triadic affiliation, we should focus on the third party. For example, we should consider if the third party shows higher levels of anxiety after perceiving the emotional state of the victim as it has been demonstrated in prairie voles under laboratory conditions^19^. Obviously, this kind of assessment is very difficult to perform under naturalistic conditions, especially in species living in large groups where numerous bystanders could become the third party affiliating with the victim.

The direct influence of social bonding and kinship on the distribution of triadic contacts could be less easily detectable in highly cohesive groups^87^ characterized by small differences between the levels of affiliation (as measured by grooming) of each dyad. Furthermore, the same argument can be applied to the quality of relationships measured by the level of relatedness, which is particularly high between gelada females belonging both to the same One-Male Unit (OMU) and even to different OMUs as demonstrated by Snyder-Mackler *et al*.^88^. For this reason, it could be extremely interesting to expand the study to wild populations in order to compare the level of reconciliation and third-party affiliation within each OMU and across different OMUs.

## Methods

### Subjects and housing

The colony of geladas, housed at the NaturZoo (Rheine, Germany), is held in two enclosures with both an indoor (a room about 36 m^2^) and outdoor facility (an island of 2,700 m^2^ surrounded by a boundary ditch). The size of the island allows the scattering of geladas and, consequently, the formation of small groups with variable composition. The enclosures are equipped with environmental enrichments allowing geladas to move freely in all three dimensions. Specifically, the outside enclosure is located in an open naturally hilly area equipped with trees, branches, ropes, and dens. The animals were fed with grass, vegetables, and pellets, which were scattered on the ground twice a day (9:30 a.m., 2:30 p.m.). Water was available *ad libitum*. No stereotypic or aberrant behaviors have been observed in this group.

Individual identification of the animals was based on sex, age, and distinctive external features (scars, size, missing fur patches, fur color, and facial traits). Thanks to the accurate birth recording made by the staff of the zoo, kinship was known. In 2007, the two OMUs were housed in the same enclosure and, in 2009, 2010 and 2011 they lived separately in two different enclosures.

This study was approved by The University of Pisa (Animal Care and Use Board). Since the study was purely observational the committee waived the need for a permit.

### Data collection

We collected behavioral data during a 6-month period in 2007 (June–November), a 4-month period in 2009 (June–September), a 2-month period in 2010 (July-August) and a 2-month period in 2011 (July-August). Data were collected through voice- and video-recorders. We gathered 1,809 hours of observations, which took place daily over 6-hr periods spanning morning (from 6:00 a.m.) and evening (until 10:00 p.m.) and all hours were equally represented.

Six observers (two per period) collected the data, with all observers being trained by the same person (the first author). The presence of at least two observers was necessary for the concurrent use of different techniques. Training was over when the observations produced a Cohen’s kappa higher than 0.80^89^. We checked for observation reliability regarding the behaviors object of this study (aggression, touching, playing, grooming, contact sitting, lip-smacking, grunting/moan, scratching) at the beginning of each month and we never obtained values below 0.80.

We defined scratching (used to measure primate anxiety)^65,66,68–70^ as a repeated movement of the hand or foot during which the fingertips are drawn across the individual’s fur. A new scratching event was assigned when the scratched body part changed or when scratching was resumed after more than 5 seconds.

Agonistic events were collected using *all-occurrences sampling*^90^. Then, after the last aggressive action of any agonistic event, we followed the victim through the *focal-animal sampling*^90^ for a 5-min post-conflict observation (PC). Matched Control focal observations (MC), were conducted the day after the conflict at the same time as the original PC and in absence of any agonistic interaction in the preceding 5 minutes^20^. Moreover, we started observing the victim for the MC when the opponents had the opportunity to socially interact (they could see each other) but were not interacting^20,91^. The rare polyadic interactions (involving >2 aggressors but only one victim) were split into dyadic components^92^. For both PCs and MCs we recorded (1) starting/ending time (minute), (2) affiliative behaviors (grooming, contact-sitting, play, touching, lip-smacking, grunting, moan) between the victim and other group members, (3) victim’s scratching (see the definition in the Result section), (4) identity of individuals interacting with the victim (5) exact moment of each interaction within the five-minute time window.

The first individual affiliating with the victim in the MCs could be every individual of the group with the exclusion of the individual who had attacked the victim in the PC (i.e., the aggressor). The selection of one specific individual as third party in the MC would produce a strong statistical bias in the evaluation of the presence of the phenomenon. For this reason, in the MC we considered as first third party each possible group mate (the previous aggressor of the corresponding PC was clearly excluded).

An aggressive event was defined as redirection when a victim attacked a group member (different from the former aggressor) within five minutes after the previous attack (PC duration).

During each focal (PC), the first affiliative contact following an aggressive event could occur between the victim and the aggressor (reconciliation), be directed by a “third party” (an individual other then the aggressor) toward the victim (spontaneous triadic party affiliation), or by the victim toward a “third party” (solicited triadic party affiliation)^28,29,36,62^. Specifically, when the bystander approached the victim and initiated the first affiliative contact interaction toward the victim the triadic party affiliation was defined as ‘spontaneous’. Instead, when the victim approached a bystander and initiated the first affiliative interaction, the triadic party affiliation was considered as ‘solicited’. The cases in which the initiator was unclear were excluded from the analysis.

For each animal we determined the number of attracted, dispersed and neutral pairs over all PC-MC pairs. In attracted pairs, affiliative contacts occurred earlier in the PC than in the MC (or they did not occur at all in the MC), whereas in dispersed pairs the affiliative contacts occurred earlier in the MC than in the PC (or they did not occur at all in the PC). In neutral pairs, affiliative contacts occurred during the same minute in the PC and the MC, or no contact occurred in either the PC or the MC.

We extracted background information on social bonding among group members using the grooming interactions recorded during focal observations other than PCs and MCs. We followed each focal subject (a single bout focal = 30 min) at different times to obtain data covering the entire day (about 6 hours of observations) in balanced proportions. Every two days all the subjects were observed at least 30 minutes.

### Definitions and data analyses

We measured the ‘Triadic Contact Tendency’ (TCT) defined as the number of attracted minus the number of dispersed pairs divided by the total number of PC–MC pairs^93^. To evaluate which behavior was preferentially used in third-party affiliation, via the same methodology we calculated TCTs for the five different behaviors involved in the post-conflict third-party affiliation: affiliative body interactions (touching/embracing), contact sitting, playing, grooming, facial expressions/vocalizations (lip-smacking/grunting + moan). The lip-smacking facial expression has an affiliative function in geladas and consists in a protrusion of the lips that are smacked together repeatedly. Grunting and moan in geladas, as it occurs for baboon species^94,95^, have an important role in the affiliation mechanisms^96,97^.

For each of the behaviours we scored attracted and dispersed pairs (see)^62^. We made use of non-parametric statistics when the data did not have a normal distribution. Each non-parametric test was performed at an individual level (each subject compared only once to avoid the problem linked to the pseudo replication of the data. To check for the presence of the post-conflict phenomena we applied the Wilcoxon signed-rank test. We used the non-parametric ANOVA Friedman’s test to compare scratching rates across three conditions: PC-no contact (absence of third-party affiliation), PC-contact (after third-party affiliation, excluding cases with reconciliation) and MC. Scratching rates during PCs-no contact were significantly higher than those during MCs in the 2–5 min time window, therefore only this period (4 minutes) was considered as relevant for the Friedman test (see Supplementary Fig. 1). Only the events not characterized by reconciliation were considered for this analysis. In case of significance across the three conditions, we ran the Dunnett’s multiple comparison test (post-hoc test) to determine which pairs of conditions differed significantly^98^. In case of multiple comparisons we adjusted the p-value according to the Bonferroni’s correction.

The analysis was performed minute-by-minute to ascertain that the decrease of the phenomena analyzed was actually due to the contact provided and not to their natural fluctuating over time after the agonistic contact^30^.

The dyadic TCT variable was normally distributed (Kruskal-Wallis, ns). Via Linear Mixed Models (used for normal distribution, LMM) we evaluated the influence of individual and relationship characteristics on TCT values. Individual characteristics included age class (adult or immature) and sex. Relationship characteristics included rank, kinship and affiliation levels. Rank was measured by using Normalized David’s Scores (Table 1). Normalized David’s scores (NDS) were calculated on the basis of a dyadic dominance index (Dij) in which the observed proportion of wins (Pij) is corrected for the chance occurrence of the observed outcome. The chance occurrence of the observed outcome is calculated on the basis of a binomial distribution with each animal having an equal chance of winning or losing in every dominance encounter^99^. The correction is necessary when, as in the case of our study groups, the interaction numbers greatly differ between dyads. We considered as kin-related those individuals belonging to grandmother/mother/offspring dyads and siblings (*r* ≥ 0.25), the others were considered non-related (non-kin). Affiliation levels were determined using grooming interactions within each dyad. Affiliation rates across dyads were arranged according to a decreasing order. We categorized the relationship quality of the dyads as strong if their grooming levels fell into the upper quartile and as medium if their grooming levels fell into the inter-quartile. All the weakly bonded dyads fell in the bottom quartile. Victim’s and third-party’s identities were entered as random factors (nominal variables) (Table 1).

We tested models for each combination involving the variables of interest, spanning from a single-variable model to a model including all the fixed factors (full model). All possible variable combinations were tested. To select the best model we used the Akaike’s Corrected Information Criterion (AICc), a measure for comparing mixed models based on the −2 (Restricted) log likelihood. The AICc corrects the Akaike’s Information Criterion (AIC) for small sample sizes. As the sample size increases, the AICc converges to AIC. The AICc allows a comparison of multiple competing models and an estimation of the best approximation of the ‘true’ process underlying the biological phenomenon under study. The model with a lower value of AICc was considered to be the best model. To quantitatively select the best model we used Akaike weights (*w*_i_). These weights provide a reliable measure of the strength of evidence for each model, and represents the ratio of delta AICc (Δ_i_) values for each model relative to the whole set of R candidate models^91^. The value of the Akaike weight (*w*_i_) indicates the probability that the model is the best among the whole set of candidate models. As suggested by Symonds and Moussalli^100^, “… models with Δ_i_ values greater than 10 are sufficiently poorer than the best AIC model as to be considered implausible” (p. 17). All tests were performed via SPSS 20.0.

## Electronic supplementary material

Video S1

Supplementary Material

Supplementary dataset

## Data Availability

Data have been submitted as Supplementary Materials.
