# Supplementary material for: High-Ranking Geladas Protect and Comfort Others After Conflicts
Source: Sci Rep. 2018 Oct 16;8:15291. doi: 10.1038/s41598-018-33548-y (PMC6191458; doi:10.1038/s41598-018-33548-y)
Supplement: Supplementary file 2 — Supplementary Material [file 41598_2018_33548_MOESM2_ESM.docx]

**HIGH-RANKING GELADAS PROTECT AND COMFORT OTHERS AFTER CONFLICTS**

**Elisabetta Palagi^1^, Alessia Leone^1^, Elisa Demuru^1^, Pier Francesco Ferrari^2^**

^1^ Museo di Storia Naturale, Università di Pisa, Via Roma 79, 56011, Calci - PISA, Italy

^2^ Institut des Sciences Cognitives Marc Jeannerod, CNRS / Université Claude Bernard Lyon
67 Bd Pinel, 69675 Bron Cedex, France

**Supplementary Table 1** - The group of geladas (*Theropithecus gelada*) housed in the NaturZoo (Rheine, Germany)

| Subjects | Year of  Birth | Mother | Sex class | Age class | Observational period |
| --- | --- | --- | --- | --- | --- |
| Gerda (OMU1)*  Gertje (OMU1)  Gitta (OMU1)  Albert (OMU1)*  Amadeus (OMU2)*  Gloria (OMU1)  Gevia (OMU1)  Gwladys (OMU2)*  Günni (OMU2)*  Hilfia (OMU1)  Angel (OMU3)  Alegria (OMU3)  Adina (OMU3)  Dominick (OMU1)  Hilfia (OMU1)  Bangle (OMU3)  Bako (OMU3)  Babs (OMU3)  Heike (OMU1)  Hera (OMU1)  Helena (OMU1) | 1978  1987  1992  1993  1994  1994  1996  1997  1997  2001  1996  1998  1998  2001  2001  2002  2003  2003  2003  2003  2004 | Unknown  Gerda  Gertje  Agathe  Afra  Gertje  Gitta  Gesa  Gertje  Gevia  Agathe  Aurora  Agathe  Buffy  Gevia  Angel  Sereba  Alegria  Gloria  Grace  Gitta | F  F  F  M  M  F  F  F  F  F  F  F  F  M  F  F  M  F  F  F  F | Adult: > 6 years | 2007  2007-2011  2007-2011  2007  2007  2007-2011  2007-2011  2007  2007  2007-2011  2009-2011  2009-2011  2009-2011  2009-2011  2007-2011  2009-2011  2009-2011  2009-2011  2007-2011  2007-2011  2007-2011 |
| Hector (OMU1)*  Hobbit (OMU1)*  Jacques (OMU2)*  Hagos (HG)  Bern (BR)  Hermine (ER)  Bounty (BO)  Belinda (BE) | 2002  2002  2003  2005  2005  2005  2005  2005 | Gitta  Gloria  Gwladys  Gloria  Adina  Gitta  Alegria  Angel | M  M  M  M  M  F  F  F | Sub-adult: 4.5–6 years | 2007  2007  2007  2007-2011  2009-2011  2007-2011  2009-2011  2009-2011 |
| Herkules (OMU1)*  Hichele (OMU1) | 2003  2007 | Gevia  Gevia | M  M | Juvenile: 2.5-4.5 years | 2007  2007-2011 |
| Jasper (OMU2)*  Tommaso  Giada  Alessia  Betta (Betta)  Davide  Dusella (DU)  Dalia (DA)  Dita (DI)  Debi (DE)  Diana (DN)  Che (CH)  Giulia (GI)  Filippa (FI)  Gaga (GA)  Alexandra | 2005  2009  2009  2009  2009  2009  2009  2009  2009-2010  2009  2010  2010  2010  2010  2010  2010 | Gwladys  Adina  Alegria  Babs  Gitta  Angel  Helena  Gloria  Hera  Grigia  Hilfia  Günni  Adina  Alegria  Hermine  Belinda | M  M  F  F  F  M  F  F  F  F  F  F  F  F  F  F | Infant: 6 months–2.5 years | 2007  2009-2011  2009-2011  2009-2011  2009-2011  2009-2011  2009-2011  2009-2011  2009-2010  2009-2011  2010-2011  2010-2011  2010-2011  2010-2011  2010-2011  2010-2011 |
| Julie (OMU1)*  AR | 2007  2011 | Günni  Hera | F  M | Black-Infant: 1-6 months | 2007  2011 |

**** Note****: OMU2 was removed from the zoo after 2007.*

**Figure S1 -** Bar graphs (mean ±SE) of the timing distribution of the renewed aggression in Post-Conflict no Contact (PCnoC; black dots) and MCs (white dots).





**Figure S2 -** Bar graphs (mean ±SE) of the timing distribution of scratching in Post-Conflict no Contact (PCnoC; black dots) and MCs (white dots).


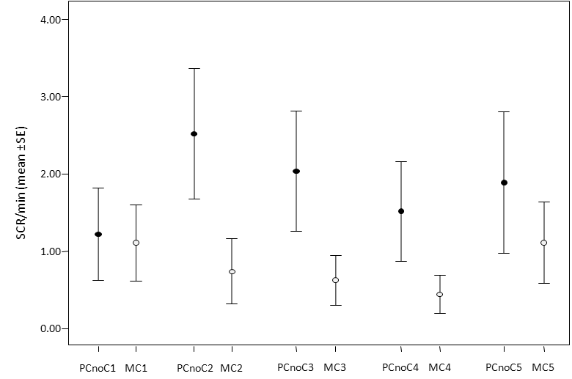


**Video S1 -** During a relax/grooming session an adult female (GL) directs an aggression against another younger adult female (ER). The aggression starts at the 7th sec and lasts 3 secs. The patterns of aggression are 1. aggressive slap 2. jaw fencing (lip retraction with mouth and teeth exposed, associated with raised eyebrows) 3. aggressive slap 4. aspirated pant. At the 8th sec the victim (ER) screams (a frightened vocalization generally associated with bared-teeth). At the 10th sec the adult female named T lip smacks and embraces the victim (ER).
